# Neurocognitive Impairments in Deficit and Non-Deficit Schizophrenia and Their Relationships with Symptom Dimensions and Other Clinical Variables

**DOI:** 10.1371/journal.pone.0138357

**Published:** 2015-09-18

**Authors:** Miao Yu, XiaoWei Tang, Xiang Wang, XiangRong Zhang, XiaoBin Zhang, WeiWei Sha, ShuQiao Yao, Ni Shu, XiangYang Zhang, ZhiJun Zhang

**Affiliations:** 1 Department of Neuropsychiatry, Affiliated ZhongDa Hospital, School of Medicine, Southeast University, Nanjing, Jiangsu, China; 2 Department of Psychiatry, Wutaishan Hospital of Yangzhou, Yangzhou, Jiangsu, China; 3 Medical Psychological Institute of the Second Xiangya Hospital, Central South University, Changsha, Hunan, China; 4 Department of Geriatric Psychiatry, Nanjing Brain Hospital Affiliated to Nanjing Medical University, Nanjing, Jiangsu, China; 5 State Key Laboratory of Cognitive Neuroscience and Learning, Beijing Normal University, Beijing, China; 6 Menninger Department of Psychiatry and Behavioral Sciences, Baylor College of Medicine, Houston, Texas, United States of America; Chiba University Center for Forensic Mental Health, JAPAN

## Abstract

**Background:**

Deficit schizophrenia (DS) has been proposed as a pathophysiologically distinct subgroup within schizophrenia. Earlier studies focusing on neurocognitive function of DS patients have yielded inconsistent findings ranging from substantial deficits to no significant difference relative to non-deficit schizophrenia patients (NDS). The present study investigated the severity and characteristic patterns of neurocognitive impairments in DS and NDS patients and their relationships with clinical variables.

**Methods:**

Attention, ideation fluency, cognitive flexibility and visuospatial memory function were assessed in 40 DS patients, 57 NDS patients, and 52 healthy controls by a comprehensive neuropsychological battery.

**Results:**

Both schizophrenia subgroups had overall more severe cognitive impairments than controls while DS performed worse on every neuropsychological measure except the Stroop interference than the NDS patients with age and education as the covariates. Profile analysis found significantly different patterns of cognitive profiles between two patients group mainly due to their differences in attention and cognitive flexibility functions. Age, education, illness duration and negative symptoms were found to have the correlations with cognitive impairments in the NDS group, while only age and the negative symptoms were correlated with the cognitive impairments in the DS group. Multiple regression analyses revealed that sustained attention and cognitive flexibility were the core impaired cognitive domains mediating other cognitive functions in DS and NDS patients respectively.

**Conclusions:**

DS patients exemplified worse in almost all cognitive domains than NDS patients. Sustained attention and cognitive flexibility might be the key impaired cognitive domains for DS and NDS patients respectively. The present study suggested the DS as a specific subgroup of schizophrenia.

## Introduction

Schizophrenia is a highly heterogeneous mental disease. The concept of deficit schizophrenia (DS) was introduced by Carpenter et al [1] to identify a clinically homogeneous subgroup of patients characterized by the presence of primary and enduring negative symptoms, which present as trait-like features of DS patients even during periods of clinical stability. DS patients differ from patients with the non-deficit form of schizophrenia (NDS) in terms of risk factors [2], gender [3–5], neuroimaging correlates [6–8], treatment response [9] and long-term clinical outcome [10, 11]. A substantial body of evidence has accumulated supporting the good test-retest and inter-rater reliability of identification of the deficit syndrome as a pathophysiologically distinct subgroup within schizophrenia [12].

To date, numerous studies focusing on the different cognitive components of DS patients have yielded a broad range of findings [13–20]. The majority of studies consistently reported that DS patients exhibited more severe impairments than NDS patients in general cognitive abilities and executive functions (assessed by WAIS-R and WCST, respectively) [15, 21] and in the ideation fluency domain (assessed by Controlled Oral Word Association Test (COWAT), Animal Naming Test (ANT) and Phonological Verbal Fluency (PVF)) [22–26]. However, previous reports on the other cognitive domains of DS patients varied considerably. For example, several studies reported a more severe deficit of cognitive flexibility in DS patients relative to NDS patients using Trail Making Test Part B (TMT-B) and Stroop interference measure [23, 24, 26–28], while discrepant results were reported from a group comparison based on the flexibility index of TMT-B minus TMT-A [11, 17] and Continuous Performance Test (CPT) [11, 17, 18, 22]. Again, although poorer competence of sustained attention [20, 24] and visuospatial memory [27, 29] in DS patients were found compared with NDS patients, some studies argued against a specific impairment in those two cognitive domains in DS patients [11, 17, 22, 25, 27]. The inconsistencies of these various reports do not allow a congruent understanding of the characteristic neurocognitive profile of DS, which might be attributed to the various clinical and methodological factors including variation of psychiatric symptoms severity, small sample size and different neuropsychological assessment tools.

Psychiatric symptoms, both positive [30, 31] and negative symptoms [32–36], and clinical variables such as age at onset [37], education [38, 39] and gender [39–41] have been proposed to relate to cognitive impairments of schizophrenia. Recently, clinical symptoms, mainly negative symptoms, were found to mediate the influence of neurocognition and social cognition on functional outcome of schizophrenia [42]. However, up to now, the relationship between neurocognitive impairments and clinical variables in DS and NDS patients has been rarely investigated. Only one study investigated a new 'chaining' habit learning task to show that more severe negative symptoms were associated with more errors on the training phase in DS patients and there were no significant correlations between frontal test measures (e.g. TMT-B, WCST and FAS) and PANSS scores in DS patients [26]. Therefore, the present study aimed to explore the characteristic patterns of neurocognitive impairments in DS and NDS patients and their relationships with clinical variables. The participants were assessed by the comprehensive neuropsychological battery measuring the domains of sustained attention, ideation fluency, cognitive flexibility and visuospatial memory function. The core cognitive domain and its mediation patterns with clinical variables were investigated in DS and NDS patients respectively with a series of statistical processes including profile analysis, Pearson correlation and the multiple regression analysis.

## Methods

### 2.1. Participants

The study was approved by the Institutional Ethical Committee for clinical research of ZhangDa Hospital Affiliated to Southeast University and written informed consent was obtained from all participants. A total of 97 clinically stable schizophrenia patients (40 DS and 57 NDS) and 52 healthy controls (HC) participated in this study. The schizophrenia patients were recruited from the psychiatric rehabilitation unit of Yangzhou Wutaishan Hospital, Jiangsu province, China. The eligibility criteria included (1) a diagnosis of schizophrenia according to Diagnostic and Statistical Manual of Mental Disorders, Fourth Edition (DSM-IV), and confirmed by the Chinese version of the Structured Clinical Interview for DSM-IV (SCID-I) [43]; (2) male right-handed Chinese Han patients and age between 20 and 65 years; (3) having stable psychiatric symptoms and antipsychotic medication for at least 12 months based on the medical record. The exclusion criteria for the patients included severe comorbid conditions, such as neurological disorders, head trauma, mental retardation, alcoholism or substance abuse and a history of previous electroconvulsive therapy. The diagnoses of deficit and non-deficit schizophrenia were reached with the help of the Chinese version of the Schedule for the Deficit Syndrome (SDS) [27]. The male healthy controls who were age- and handedness-matched with the patients were recruited from the local community meeting the following criteria: (1) no lifetime history of psychotic, mood, and substance abuse or dependence, ascertained by the Structured Clinical Interview for DSM-IV Non-Patient version (SCID-NP) [44]; (2) no history of organic brain disorder, mental retardation, or severe head trauma; and (3) no family history of psychiatric disorders.

### 2.2. Clinical and neuropsychological assessment

#### 2.1.1. Clinical evaluation

After obtaining demographic and medication information, the deficit syndrome was determined according to the Schedule for Deficit Syndrome (SDS) [45], a semi-structured interview by two independent psychiatrists (XBZ and XWT) that achieved a high inter-rater reliability [intraclass correlation coefficient (ICC) = 0.86]. The SDS rated the deficit syndrome as present if two of the following symptoms (restricted affect, diminished emotional range, poverty of speech, curbing of interests, diminished sense of purpose and diminished social drive) had been at least moderately severe, persistent over 12 months and not attributable to secondary sources (e.g., medication side effects, depression, paranoia, and anxiety). Subsequently, total SDS score and the two factors (Factor 1: avolition and Factor 2: poor emotional expression) were calculated [46, 47]. The severity of schizophrenic symptoms was evaluated by the Brief Psychiatric Rating Scale (BPRS), the Scale for the Assessment of Negative Symptoms (SANS), and the Scale for the Assessment of Positive Symptoms (SAPS). BPRS scales were organized into separate positive, negative, disorganized and affect syndromes based on the findings of the most comprehensive factor analysis of the 18-item BPRS [48, 49]. Clinical and demographic data were summarized in Table 1.

**Table 1:** Demographics and clinical characteristics for DS, NDS and HC groups.

	DS (n = 40)	NDS (n = 57)	HC (n = 52)	*F*/χ2/*t*	*p*
Age (years)	49.38±7.29	46.14±7.18	45.81±8.77	2.795	0.064
Education (years)	8.98±1.97^△^	8.91±1.85^#^	10.42±2.75	7.513	0.001
Age at Onset (years)	21.80±2.92	22.35±2.49		-0.999	0.320
Duration of illness (years)	27.58±6.89*	23.79±7.19		2.596	0.011
BPRS total score	31.88±2.86**	27.49±2.76		7.591	<0.001
Positive syndrome	6.23±1.19	6.40±1.16		-0.738	0.462
Negative syndrome	12.30±1.62**	7.49±1.00		16.665	<0.001
Disorganized syndrome	6.48±1.01	6.54±0.97		-0.339	0.735
Affect	6.88±1.11	7.05±1.27		-0.711	0.479
SANS total score	56.35±8.35**	32.30±6.57		15.861	<0.001
SAPS total score	9.20±3.92	10.19±5.06		-1.042	0.300
SDS total score	10.95±2.42**	4.16±2.35		13.844	<0.001
avolition	5.88±1.62 **	2.47±1.42		10.972	<0.001
poor emotional expression	5.08±1.10 **	1.68±1.17		14.442	<0.001
Smoking ratio (%)	65.00	70.20		0.290	0.591
Antiparkinson agent ratio (%)	42.5	35.1		0.547	0.459
Benzodiazepine agent ration (%)	20.0	15.8		0.288	0.591
CPZ-equivalent daily dose (mg/day)	496.88±217.54	506.84±211.08		-0.226	0.822

Note: DS: deficit schizophrenia; NDS: non-deficit schizophrenia; HC: healthy controls; BPRS: Brief Psychiatric Rating Scale; SANS: the Scale for the Assessment of Negative Symptoms; SAPS: the Scale for the Assessment of Positive Symptoms; SDS: schedule for the deficit syndrome; CPZ: chlorpromazine;

** *p* <0.001 DS vs. NDS;

* *p* <0.05 DS vs. NDS;

^△^ *p* <0.05 DS vs. HC;

^#^ *p* <0.05 NDS vs. HC.

#### 2.2.2. Neuropsychological assessment

A battery of classical neuropsychological tests that consisted of Digit Vigilance test (DVT), Animal Naming Test (ANT), Controlled Oral Word Association Test (COWAT), Block Design (Wechsler adult intelligence scale- Chinese Revision WAIS-RC), Trail Making Test-A, B (TMT-A,B), Stroop Color-Word test (SCWT) and Spatial Processing (Block design) was used. The cognitive variables were further grouped into the four rationally motivated domains as sustained vigilance/attention (hereinafter labeled as sustained attention), cognitive flexibility, ideation fluency and visuospatial memory, based on previous reports regarding cognitive processes assessed by each of the tasks (Tables 2 and 3) [24, 50–53].

**Table 2:** Comparisons of raw neuropsychological performance among DS, NDS and HC groups.

	DS (n = 40)	NDS (n = 57)	HC (n = 52)	*F*	*p*
Animal Naming test	9.57±3.13* ^△△^	12.33±4.38 ^##^	17.62±4.59	37.486	<0.001
COWAT	4.95±3.05* ^△△^	6.95±3.67^#^	8.94±2.29	15.117	<0.001
Digit vigilance test (seconds)	285.77±121.60** ^△△^	187.58±94.14^#^	135.49±39.74	25.490	<0.001
TMT-A (seconds)	131.28±69.45** ^△△^	77.04 ±32.86^#^	48.77±21.19	34.665	<0.001
TMT-B (seconds)	283.76±145.70** ^△△^	196.99±55.11^##^	119.11±60.25	29.309	<0.001
Stroop words only	45.28±20.00* ^△△^	59.28±17.62^##^	78.92±16.10	33.364	<0.001
Stroop colors only	27.40±12.81* ^△△^	36.56±11.29^##^	50.63±13.89	30.984	<0.001
Stroop interference	17.78±10.22^△△^	21.82±8.62^##^	32.06±10.17	21.383	<0.001
Spatial processing (Block design)	11.58±4.54* ^△△^	13.79 ±3.48^##^	17.63±3.36	23.293	<0.001
WAIS-RC (Block Design)	12.88±8.93** ^△△^	21.91±7.02^##^	28.52±8.22	35.423	<0.001

Note: COWAT: Controlled Oral Word Association test; TMTA&B: Trail Making Tests A&B; WAIS-RC: Wechsler adult intelligence scale (Chinese version); DS: deficit schizophrenia; NDS: non-deficit schizophrenia; HC: healthy controls;

** *p* <0.001 DS vs. NDS;

* *p* <0.05 DS vs. NDS;

^△△^ *p* <0.001 DS vs. HC;

^##^ *p* <0.001 NDS vs. HC;

^#^ *p* <0.05 NDS vs. HC.

**Table 3:** Descriptive statistics of domain-specific composite scores, effect sizes and Cronbach’s alpha for each cognitive domain.

Cognitive domain	DS (n = 40)	NDS (n = 57)	Effect size (Cohen’s *d*)	Cronbach's Alpha
Sustained vigilance/Attention	-11.44±6.65	-4.88±4.25	1.176	0.803
Ideation fluency	-3.50±1.74	-2.02±2.22	0.742	0.673
Cognitive flexibility	-4.14 ±2.97	-2.30±1.45	0.787	0.661
Visuospatial memory	-3.71±1.92	-1.95±1.53	1.014	0.648

Note: Patients’ neuropsychological test scores were standardized using the healthy control group data. Sustained vigilance/attention domain includes Stroop words only and colors only, Trail making test part A and Digit vigilance test. Ideation fluency domain includes Controlled Oral Word Association test and Animal Naming Test. Cognitive flexibility includes Stroop color/word interference test and Trail making test part B. Visuospatial memory domain includes Spatial processing test and Wechsler adult intelligence scale (Block Design, Chinese version). DS: deficit schizophrenia; NDS: non-deficit schizophrenia.

#### 2.2.3. Procedure

The diagnostic information was collected using the SCID and SDS interviews after the participants completed the informed consent and demographics form. Following the interview, the neuropsychological tests were administered individually in a small room, with minimal extraneous stimulation. These tests were assessed under the standard instructions and scoring methods, which required approximately 90 min to administer. All tests were administered to the subjects in the same order and by the same investigators who were unaware of the subjects' status at the time of administration and scoring. Psychiatric assessments such as the BPRS, SANS, and SAPS were completed on the same day.

### 2.3. Data analysis

The SPSS 13.0 was used for statistical analyses. Data of demographic, clinical and cognitive variables are presented as mean ± standard deviation (S.D.). Continuous and categorical variables were analyzed by the ANCOVA analysis and the chi-square test respectively. Psychiatric symptoms between DS and NDS groups were compared using t-tests.

To combine the neuropsychological variables, the standardized *Z* scores of each individual test, being created by using control group data across all patients, were summed to calculate the cognitive domain values. Variables in which good performance was represented by lower values (e.g. TMT) were adjusted for sign to ensure that higher *Z*-scores represented better performance for all variables. Cronbach’s alpha and Cohen’s d effect sizes [54, 55] were computed for each domain. The profile analysis [56] was performed to compare the mean *Z*-scores for each neuropsychological test and cognitive domain with age, education, illness duration and drug dose as covariates in order to compare the patterns of neurocognitive impairments between the two subgroups. Pearson correlation was analyzed between neurocognitive domain and clinical variables in two patient groups respectively. The multiple linear regression analysis was further used to determine whether deficits of several cognitive domains are mediated by a certain core cognitive domain in two subgroups. The significance level was set at *p*<0.05.

## Results

### 3.1. Demographic and clinical characteristics

Demographic and clinical characteristics for the subjects are presented in Table 1. The ANOVA analysis showed significant differences in education (*F* = 7.513, *p* = 0.001) but not age (*F* = 2.795, *p* = 0.064) among the three groups. LSD post hoc comparisons revealed shorter education periods for DS (*p* = 0.002) and NDS (*p* = 0.001) patients relative to HC subjects, while the two patient subgroups did not differ significantly (*p* = 0.892). Chi-square and T-tests results indicated that DS and NDS patients had no significant differences in the mean age of onset, smoking, the usage of antiparkinson agents or benzodiazepine agents and antipsychotic medicine dosage (chlorpromazine equivalents) except for a longer illness duration (*p* = 0.011) in the DS group. There was no significant difference (*χ* ^2^ = 1.106, *p* = 0.575) in the type of antipsychotic treatment between the DS and NDS groups (conventional antipsychotics: 45.0% [n = 18] and 35.1% [n = 20]; novel antipsychotics: 32.5% [n = 13] and 35.1% [n = 20]; combination: 22.5% [n = 9] and 29.8% [n = 17], respectively). The DS patients showed more severe psychopathological total symptom and negative symptoms (all *p*<0.001) than NDS but not in either positive, affect or disorganized syndrome (all *p*>0.300).

### 3.2. Neuropsychological evaluation

The general linear model (GLM) analysis revealed significant overall differences among the three groups for every individual neuropsychological measure with age and education as covariates (all *p*<0.001, Table 2). Post hoc comparisons confirmed that both schizophrenia subgroups performed significantly worse than the control group on every cognitive measure (all *p*<0.05). Furthermore, the DS group showed greater impairment in most neuropsychological measures compared with the NDS group (all *p*<0.05) except the Stroop interference (*p* = 0.148). The Cronbach's alpha for the four cognitive domains ranged from 0.648 to 0.803 indicating relatively high internal consistency among the measures (Table 3). We calculated Cohen’s *d* effect size estimation of the performance difference using the *Z*-transformed cognitive domain scores between the schizophrenia subgroups for the four cognitive domains (Table 3). The effect sizes for DS vs. NDS ranged from *d* = 0.742 to 1.176 across four cognitive domains indicating that the DS-NDS differences of all the cognitive domains were significant and achieved moderate to large effect sizes.

### 3.3. Profile analysis of cognitive function in DS and NDS patients

**Fig 1:**
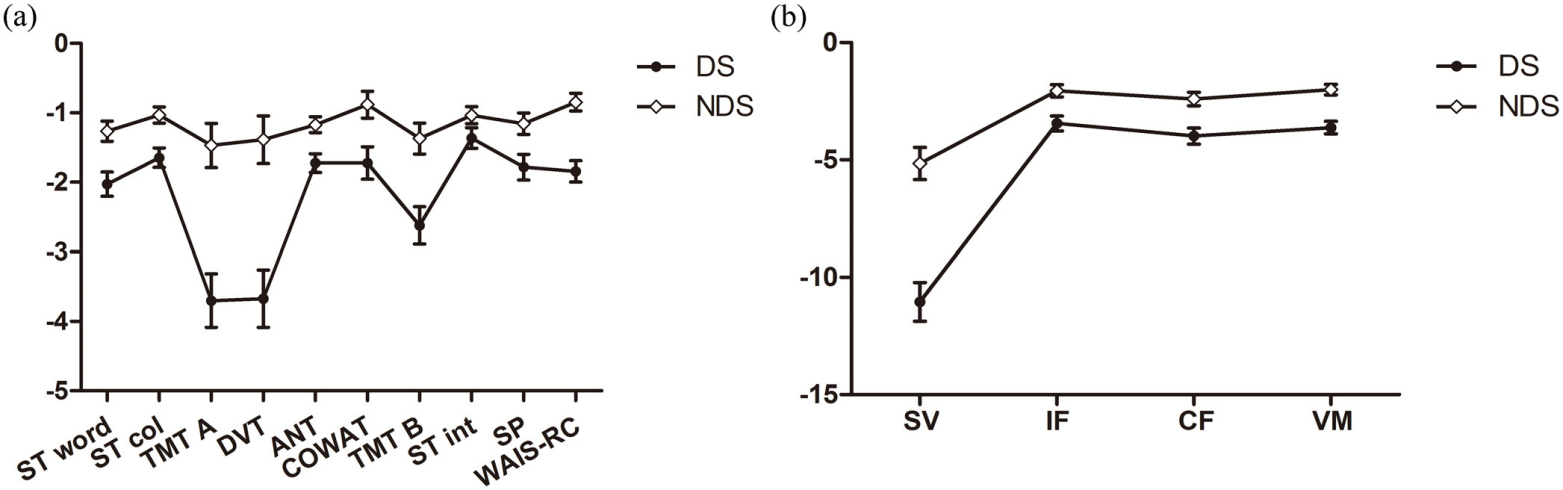
Profile analysis for neuropsychological tests and cognitive domains of DS and NDS groups. Note: Profile analysis for (a) neuropsychological tests and (b) cognitive domains of DS and NDS groups. ST word: Stroop word only; ST col: Stroop color only; TMTA&B: Trail Making Tests A&B; DVT: Digit vigilance test; ANT: Animal Naming test; COWAT: Controlled Oral Word Association test; ST int: Stroop interference test; SP: Spatial processing (Block Design); WAIS-RC: Wechsler adult intelligence scale (Block Design, Chinese version); SV: Sustained vigilance/attention; IF: Ideation fluency; CF: Cognitive flexibility; VM: Visuospatial memory. The error bars on Fig 1 was standard errors.

As shown in Fig 1a, the profile analysis revealed a significant diagnosis effect (*F* (9, 83) = 2.218; *p* = 0.029) on the pattern of cognitive profiles for the ten cognitive test variables. The DS group showed relatively greater impairment in TMT-A, Digit vigilance test and TMT-B than the other cognitive tests, which led to the steeper slopes for the line adjacent to those three cognitive tests in the DS group relative to those of the NDS group. DS and NDS group did not differ significantly in form or pattern of cognitive profiles (*F* (6, 86) = 1.503; *p* = 0.187) when the data of TMT-A, Digit vigilance test and TMT-B were removed from profile analysis. Moreover, the effect of diagnostic subtype on the cognitive domain profiles was also significant (*F* (3, 89) = 8.348; *p*<0.001) (Fig 1b). The lines linked with sustained attention and cognitive flexibility had steeper slopes in the DS group compared to those of the NDS group. This suggested that the significant difference of cognitive profile between two patient groups was attributable to those two cognitive domains: sustained attention mainly assessed by TMT-A and Digit vigilance test and cognitive flexibility mainly assessed by TMT-B in the present study.

### 3.4. Relationships between clinical features and cognitive domains in DS group

Pearson correlation showed that sustained attention function was significantly correlated with the other three cognitive domains while the cognitive flexibility was also correlated with visuospatial memory (Table A in S1 Table). We then examined the correlations between the clinical variables and performance in the cognitive domains (Table A in S2 Table). Most clinical variables such as age at onset, education, duration of illness, CPZ-equivalent dose, BPRS and SAPS total score did not correlate significantly with four cognitive domains. However, age was negatively correlated with sustained attention (*p* = 0.033) and the SANS total score was found to have negative correlations with the four cognitive domains (*p*<0.05) in the DS patient group. Finally, the correlation between age and SANS total score was further analyzed, showing significantly positive correlation between them (*r* = 0.314, *p* = 0.049).

Partial correlation analysis was performed to determine the relationship between age, SANS and the sustained attention function. Age was no longer significantly correlated with the sustained attention domain (*p* = 0.179) when the SANS total score was included as a covariate. This indicated that the relationship of age to the sustained attention function was fully mediated by the negative symptom. Then a series of regression models were developed to determine whether the negative symptom rather than age could predict cognitive performance in DS patients. As shown in Table A in S3 Table, the negative symptom contributed independently to different cognitive domains.

Based on the results in the profile analysis, we hypothesized that either sustained attention or the cognitive flexibility function held the key position in the pattern of cognitive impairments in the DS group. A series of hierarchical regressions were performed to determine whether sustained attention or cognitive flexibility mediated the association between clinical variables and the other cognitive domains in DS patients. Either sustained attention or cognitive flexibility was entered in step one, and the SANS total score was entered one each time in step two, while one of the left three cognitive domains was selected as dependent variable. If changes in other cognitive domains are mediated through the core impairment cognitive domain, then the clinical variable (SANS total score) should no longer be a significant predictor once we account for the core impairment cognitive domain-related variance.

**Fig 2:**
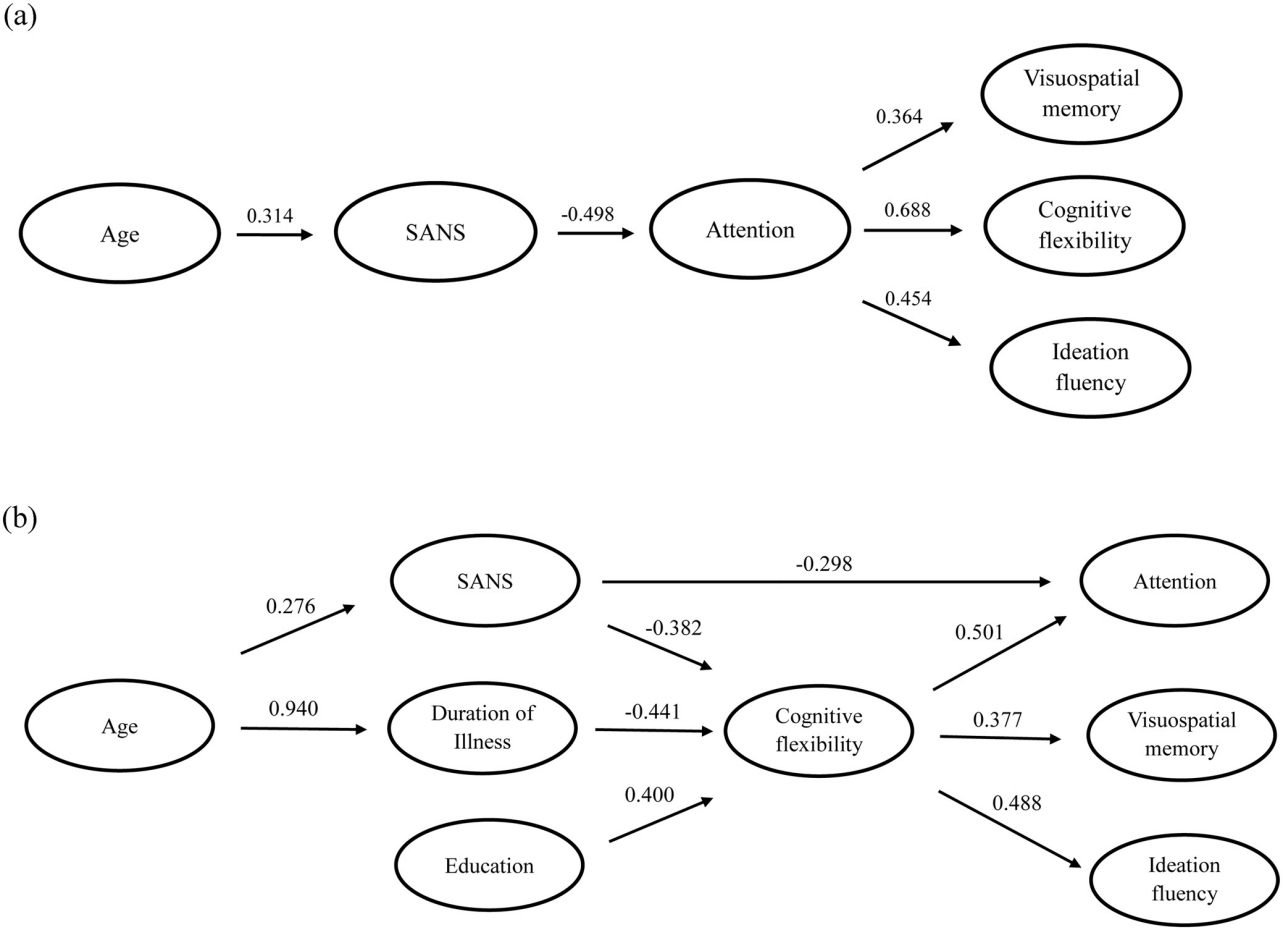
The multiple regression analyses for relationships between clinical features and cognitive domains in DS and NDS group, respectively. Note: The relationships between clinical variables and cognitive domains impairments in (a) DS group and (b) NDS group were carried out with the hierarchical regression analyses. The numerical values represent standardized beta weights.

When selected as the core cognitive domain and entered in step one, sustained attention was survived in all regression analyses that the cognitive flexibility (*β* = 0.688, *p*<0.001), ideation fluency (*β* = 0.454, *p* = 0.003) and visuospatial memory (*β* = 0.364, *p* = 0.021) was selected as the dependent variables respectively, while the SANS score lost the contributions to cognitive flexibility, ideation fluency and visuospatial memory respectively (*p*>0.096). It indicated that sustained attention had a full mediation effect among the negative symptom and the three cognitive domains. However, when cognitive flexibility was taken into account as the core impairment domain, the negative symptom also had the significant contribution to sustained attention (cognitive flexibility, *β* = 0.583, *p*<0.001; SANS, *β* = -0.280, *p* = 0.025), indicating a partial mediation effect of cognitive flexibility. In addition, the cognitive flexibility was not a significant factor in the regression analysis between the negative symptom and ideation fluency (*β* = 0.245, *p* = 0.127), indicating no mediation effect of cognitive flexibility among them. Therefore, these regression analyses in coordination with the profile analysis indicated that sustained attention had a core role in determining the pattern of cognitive impairments in the DS group (as illustrated in Fig 2a).

### 3.5. Relationships between clinical features and cognitive domains in NDS group

#### 3.5.1. Correlation among cognitive domains, demographic and clinical variables

Firstly, Pearson correlation demonstrated that all the cognitive domains were significantly intercorrelated in the NDS group (*p*<0.05) (Table B in S1 Table).

Secondly, we then examined which demographic and clinical variables were correlated with cognitive domains. As shown in Table B in S2 Table, sustained attention was negatively correlated with age (*p* = 0.011), illness duration (*p* = 0.029) and the SANS total score (*p*<0.001) respectively, while sustained attention had a positive correlation with education (*p* = 0.035). Ideation fluency had a positive and negative correlation with education (*p* = 0.046) and the negative symptom (*p* = 0.007) respectively. Cognitive flexibility was negatively correlated with age (*p* = 0.005), illness duration (*p* = 0.001) and the negative symptom (*p* = 0.003), and positively correlated with education (*p* = 0.002). Visuospatial memory was positively correlated with education (*p* = 0.049) and negatively correlated with the negative symptom (*p* = 0.047). Thirdly, the correlations between these clinical variables showing significant correlation with cognitive domains were further analyzed. Only age was significantly correlated with illness duration (*r* = 0.940, *p*<0.001) and the SANS total score (*r* = 0.276, *p* = 0.038). The education factor was not correlated with any other demographic and clinical variables.

#### 3.5.2. Identification of the contribution of clinical variables to cognitive domains

A series of hierarchical regressions were performed to determine the relationships among age, illness duration and the negative symptom. Age was no longer significantly contributed to performance in any of the cognitive domains (*p*>0.147) when the duration of illness and the SANS total score were taken into account. Thus, the relationship of age to cognitive function was fully mediated by illness duration and the negative symptom, which illustrated that illness duration and the negative symptom, but not age, may make independent contributions to cognitive performance.

The regression models were then developed to determine whether education, illness duration and the negative symptom except age could predict cognitive performance in NDS patients. As shown in Table B in S3 Table, the above three clinical variables (e.g. education, illness duration and the negative symptom) contributed independently to different cognitive domains.

#### 3.5.3. Identification of the core cognitive domain in the NDS patients

The profile analysis indicated that either sustained attention or cognitive flexibility could be the basic cognitive domain in NDS patients. A series of hierarchical regressions were also performed to determine whether sustained attention or cognitive flexibility mediated the association between clinical variables and the other cognitive domains in NDS patients. Either sustained attention or cognitive flexibility was entered in step one, and one of the above three clinical variables was entered one each time in step two, while one of the left three cognitive domains was selected as dependent variables (S4 Table). When selected as the core cognitive domain and entered in step one, cognitive flexibility was survived in all regression analyses (*p*<0.004). Education no longer predicted performance in sustained attention (*β* = 0.041, *p* = 0.730), ideation fluency (*β* = 0.084, *p* = 0.519) and visuospatial memory (*β* = 0.132, *p* = 0.337) after variance associated with cognitive flexibility was taken into account. Illness duration lost the contribution to sustained attention (*β* = -0.022, *p* = 0.857) and the negative symptom also lost the contribution to ideation fluency (*β* = -0.197, *p* = 0.123) and visuospatial memory (*β* = -0.141, *p* = 0.300) respectively. It indicated that cognitive flexibility had a full mediation effect among them. However, the negative symptom contributed to sustained attention (cognitive flexibility, *β* = 0.501, *p*<0.001; SANS, *β* = -0.298, *p* = 0.008), indicating a partial mediation effect of cognitive flexibility to sustained attention.

When sustained attention was taken into account as the core cognitive domain, education (*β* = 0.247, *p* = 0.024) and illness duration (*β* = -0.288, *p* = 0.008) continued to significantly predict the cognitive flexibility, indicating the partial mediation effects of sustained attention between clinical variables and the cognitive flexibility. In addition, the regression model was not significant (*F* (2, 54) = 2.768; *p* = 0.072) when the visuospatial memory was the dependent variable and the negative symptom was entered as step two, indicating no mediation effect of sustained attention between the negative symptom and visuospatial memory. Therefore, cognitive flexibility rather than sustained attention was suggested as the core cognitive domain in the NDS patients. The relationships between the clinical variables and cognitive domains in NDS group are illustrated in Fig 2b.

## Discussion

The present results found that both schizophrenia subgroups performed worse than the control group on every cognitive measure and cognitive domain. DS patients showed greater impairments on almost every neuropsychological measure than NDS patients except the Stroop interference test. The present results are consistent with past studies suggesting that DS patients had greater impairment on each measure of ideation fluency and cognitive flexibility domains (e.g. TMT-B, COWAT and Animal Naming Test) [22–28], which are both critical roles of executive function. In addition, the present study found that DS patients performed worse than NDS patients on sustained attention (assessed in Stroop words and colors only, TMT-A and DVT) and visuospatial memory (assessed in Spatial processing and WAIS-RC) tasks. Several previous studies examining those two cognitive domains reported inconsistent results ranging from substantial decline in DS patients [18, 20, 24, 27, 28, 48] to no significant difference between two patients groups [11, 17, 22, 25]. Furthermore, the profile analysis identified significantly different patterns of cognitive profiles between DS and NDS patients group, which was especially attributed to distinct differences in sustained attention (TMT-A and DVT) and cognitive flexibility (TMT-B) functions between two patient groups. Cascella et al. (2008) [22] reported no robust different patterns of cognitive function derived from the profile analysis. These inconsistent results might be due to the aforementioned clinical or methodological confounders including sample size, social environment factor, and different assessment tools [11, 22, 23, 25].

The present study provided the first evidence that DS patients are characterized by a sustained attention deficit as the core impaired cognitive domain, mediating the negative symptoms on the other three cognitive domains. Consistently, previous studies found that schizophrenia patients with persistent negative symptoms displayed more severe sustained attention impairment in term of TMT-A test [36, 57] or other relative tests [58]. A recent independent empirical study and meta-analysis also pointed out that DS patients may show global cognitive impairments with especially significantly poorer performance on TMT-A test indicating the particular impairment of attention sustainment in DS patients [48]. Furthermore, the worse performance in processing speed and attention function has been reported in the first-episode drug naïve patients with deficit syndrome than in equivalent patients without deficit syndrome [58]. Meanwhile, the present study found that cognitive flexibility was the core impaired cognitive domain for NDS but not DS patients, as cognitive flexibility was shown to mediate the influence of other independent contributors, such as education, duration of illness and the negative symptoms, on the other three cognitive domains in NDS patients. It has been demonstrated that there may be different neural circuitries underlying the diverse components of cognitive function. Sustained attention assessed by measurements such as TMT-A, Stroop words and colors only, and DVT, is regarded as a non-specific lobe-based assignment [48], while impairment of cognitive flexibility, a component of executive function and assessed by TMT-B, Stroop interference test and continuous performance test, is associated with dysfunction of frontal-striatal regions [18, 48, 59, 60]. Recent neuroimaging studies reported that both DS and NDS groups had structural deficits such as smaller dorsolateral prefrontal cortex [8, 61] and temporal lobes [8] relative to healthy subjects. However, abnormalities of frontal-striatal regions in DS were generally not greater than those of NDS patients [8, 12], whereas the dorsolateral prefrontal gray matter volume reduction was even more severe in NDS patients [61, 62]. Interestingly, apart from frontoparietal dysfunction [63], DS patients were consistently reported to have reduced temporal gray [8, 64–66] and white matter volume [66] compared with NDS patients. This evidence for differential structural deficits might provide a possible explanation for the distinction of a core impaired cognitive domain between DS and NDS patients.

The present study found that the SANS total score was negatively correlated with different cognitive domains in both DS and NDS patients, which was consistent with the most previous studies recruiting undifferentiated schizophrenia patients [41, 42, 67]. Importantly, the present study firstly reported that negative syndrome mediated the distinct core cognitive domain with the other cognitive domains in DS and NDS patients respectively. Additionally, education and illness duration were correlated with performance of cognitive domains in the NDS group rather than the DS group. Illness duration may represent the potential progressive impairment of repeated psychotic episodes on cognitive function in NDS patients [12]. Education, as is well-known, might possibly possess the protective effect on cognitive dysfunction in NDS patients. These correlations implied the possibility of an interaction between cognitive impairments and environmental factors in NDS patients. Nevertheless, the DS patients demonstrate fewer interactions with other clinical variables, which converges with the concept described by Carpenter et al that the deficit syndrome is primary “trait-like” [1]. The disparate mediation pattern of clinical variables and the core impaired cognitive domains supports the hypothesis of the deficit syndrome as a pathophysiologically distinct subgroup within schizophrenia.

Certain limitations and methodological issues of the study should be considered when interpreting the results. Firstly, since all the patients received antipsychotic treatment and partially received the antiparkinson or benzodiazepine agents, it is conceivable that the cognitive side effect of these medications might contribute to the impairment of cognitive function, although the type and dosage of antipsychotic drug and the percentage use of these agents had been well controlled between the two patient subgroups. Future studies could further explore the relationships in regard to the use of the medications and the cognitive functions in the DS patients. Secondly, chronic schizophrenia patients were recruited in the present study to guarantee the status of clinical stability as a requirement of DS categorization. The illness duration in patients with schizophrenia might import the potential confounders for cognitive comparison. The present study attempted to restrict variance due to confounders including gender, fluctuations of psychiatric symptom and social environment. Future studies ought also to consider out-patients, female samples and larger sample sizes. Furthermore, general cognitive function, certain clinical variables such as the premorbid IQ and certain neuropsychological processes were not assessed in this study. For example, verbal memory and social cognition might be important influences on the functional outcome in schizophrenia [68, 69]. Deficit patients have also been shown to have abnormal visual information-processing impairments [70] and aberrant attention bias for negative information [71, 72]. This study did not provide results in these respects and might be biased by the lack of these measures.

In summary, the present findings have provided evidence of both the qualitative and quantitative differences in neurocognitive functions between DS and NDS patients. More severe impairments of almost all individual neuropsychological measures and cognitive domains were found in DS than NDS patients. Sustained attention and cognitive flexibility might possibly be the core impaired cognitive domains for DS and NDS patients respectively. The present study emphasized that the deficit syndrome might be a specific subgroup within schizophrenia, which warrants further investigation of its underlying morphometric brain features and etiopathogenic mechanisms.

## Supporting Information

S1 Table Pearson correlation analyses among cognitive domains in DS (Table A) and NDS group (Table B), respectively. Note: **p<0.001; * *p*<0.05.
(DOCX)

S2 Table Pearson correlation analyses between clinical features and cognitive domains in DS (Table A) and NDS group (Table B), respectively. Note: * *p*<0.05.
(DOCX)

S3 Table Linear regression analyses for independent contributions of clinical variables to cognitive function in DS (Table A) and NDS group (Table B), respectively. Note: **p<0.001; * *p*<0.05.
(DOCX)

S4 Table Comparison of the mediator role of cognitive flexibility (Table A) and attention function (Table B) between clinical variable and the other cognitive domains in NDS group. NOTE: The regression analysis was only performed in the variables correlated with the cognitive functions.
(DOCX)

## References

[1] Carpenter WT Jr, Heinrichs DW, Wagman AM. Deficit and nondeficit forms of schizophrenia: the concept. Am J Psychiatry. 1988;145(5):578–83. 3358462 10.1176/ajp.145.5.578

[2] Messias E, Kirkpatrick B, Bromet E, Ross D, Buchanan RW, Carpenter WT Jr, et al Summer birth and deficit schizophrenia: a pooled analysis from 6 countries. Arch Gen Psychiatry. 2004;61(10):985–9. 15466671 10.1001/archpsyc.61.10.985

[3] Kirkpatrick B, Ross DE, Walsh D, Karkowski L, Kendler KS. Family characteristics of deficit and nondeficit schizophrenia in the Roscommon Family Study. Schizophr Res. 2000;45(1–2):57–64. 10978873 10.1016/s0920-9964(99)00164-4

[4] Arango C, Bobes J, Kirkpatrick B, Garcia-Garcia M, Rejas J. Psychopathology, coronary heart disease and metabolic syndrome in schizophrenia spectrum patients with deficit versus non-deficit schizophrenia: findings from the CLAMORS study. Eur Neuropsychopharmacol. 2011;21(12):867–75. 10.1016/j.euroneuro.2011.03.005 21477998

[5] Roy MA, Maziade M, Labbe A, Merette C. Male gender is associated with deficit schizophrenia: a meta-analysis. Schizophr Res. 2001;47(2–3):141–7. 11278131 10.1016/s0920-9964(99)00231-5

[6] Lahti AC, Holcomb HH, Medoff DR, Weiler MA, Tamminga CA, Carpenter WT Jr. Abnormal patterns of regional cerebral blood flow in schizophrenia with primary negative symptoms during an effortful auditory recognition task. Am J Psychiatry. 2001;158(11):1797–808. 11691685 10.1176/appi.ajp.158.11.1797

[7] Quarantelli M, Larobina M, Volpe U, Amati G, Tedeschi E, Ciarmiello A, et al Stereotaxy-based regional brain volumetry applied to segmented MRI: validation and results in deficit and nondeficit schizophrenia. Neuroimage. 2002;17(1):373–84. 12482090 10.1006/nimg.2002.1157

[8] Galderisi S, Quarantelli M, Volpe U, Mucci A, Cassano GB, Invernizzi G, et al Patterns of structural MRI abnormalities in deficit and nondeficit schizophrenia. Schizophr Bull. 2008;34(2):393–401. 17728266 10.1093/schbul/sbm097 PMC2632416

[9] Kirkpatrick B, Buchanan RW, Ross DE, Carpenter WT Jr. A separate disease within the syndrome of schizophrenia. Arch Gen Psychiatry. 2001;58(2):165–71. 11177118 10.1001/archpsyc.58.2.165

[10] Tek C, Kirkpatrick B, Buchanan RW. A five-year followup study of deficit and nondeficit schizophrenia. Schizophr Res. 2001;49(3):253–60. 11356586 10.1016/s0920-9964(00)00146-8

[11] Galderisi S, Bucci P, Mucci A, Kirkpatrick B, Pini S, Rossi A, et al Categorical and dimensional approaches to negative symptoms of schizophrenia: Focus on long-term stability and functional outcome. Schizophr Res. 2013;147(1):6. 10.1016/j.schres.2013.03.020 23608244

[12] Galderisi S, Maj M. Deficit schizophrenia: an overview of clinical, biological and treatment aspects. Eur Psychiatry. 2009;24(8):493–500. 10.1016/j.eurpsy.2009.03.001 19553087

[13] Buchanan RW, Strauss ME, Kirkpatrick B, Holstein C, Breier A, Carpenter WT Jr. Neuropsychological impairments in deficit vs nondeficit forms of schizophrenia. Arch Gen Psychiatry. 1994;51(10):804–11. 7944870 10.1001/archpsyc.1994.03950100052005

[14] Seckinger RA, Goudsmit N, Coleman E, Harkavy-Friedman J, Yale S, Rosenfield PJ, et al Olfactory identification and WAIS-R performance in deficit and nondeficit schizophrenia. Schizophr Res. 2004;69(1):55–65. 15145471 10.1016/S0920-9964(03)00124-5

[15] Bryson G, Whelahan HA, Bell M. Memory and executive function impairments in deficit syndrome schizophrenia. Psychiatry Res. 2001;102(1):29–37. 11368837 10.1016/s0165-1781(01)00245-1

[16] Tiryaki A, Yazici MK, Anil AE, Kabakci E, Karaagaoglu E, Gogus A. Reexamination of the characteristics of the deficit schizophrenia patients. Eur Arch Psychiatry Clin Neurosci. 2003;253(5):221–7. 14504990 10.1007/s00406-003-0434-5

[17] Galderisi S, Maj M, Mucci A, Cassano GB, Invernizzi G, Rossi A, et al Historical, psychopathological, neurological, and neuropsychological aspects of deficit schizophrenia: a multicenter study. Am J Psychiatry. 2002;159(6):983–90. 12042187 10.1176/appi.ajp.159.6.983

[18] Buchanan RW, Strauss ME, Breier A, Kirkpatrick B, Carpenter WT Jr. Attentional impairments in deficit and nondeficit forms of schizophrenia. Am J Psychiatry. 1997;154(3):363–70. 9054784 10.1176/ajp.154.3.363

[19] Horan WP, Blanchard JJ. Neurocognitive, social, and emotional dysfunction in deficit syndrome schizophrenia. Schizophr Res. 2003;65(2–3):125–37. 14630305 10.1016/s0920-9964(02)00410-3

[20] Cohen AS, Docherty NM. Deficit versus negative syndrome in schizophrenia: prediction of attentional impairment. Schizophr Bull. 2004;30(4):827–35. 15954193 10.1093/oxfordjournals.schbul.a007135

[21] Polgar P, Rethelyi JM, Balint S, Komlosi S, Czobor P, Bitter I. Executive function in deficit schizophrenia: what do the dimensions of the Wisconsin Card Sorting Test tell us? Schizophr Res. 2010;122(1–3):85–93. 10.1016/j.schres.2010.06.007 20627227

[22] Cascella NG, Testa SM, Meyer SM, Rao VA, Diaz-Asper CM, Pearlson GD, et al Neuropsychological impairment in deficit vs. non-deficit schizophrenia. J Psychiatr Res. 2008;42(11):930–7. 18021807 10.1016/j.jpsychires.2007.10.002

[23] Farkas M, Polgár P, Kelemen O, Réthelyi J, Bitter I, Myers CE, et al Associative learning in deficit and nondeficit schizophrenia. Neuroreport. 2008;19(1):55–8. 10.1097/WNR.0b013e3282f2dff6 18281892

[24] Rethelyi JM, Czobor P, Polgar P, Mersich B, Balint S, Jekkel E, et al General and domain-specific neurocognitive impairments in deficit and non-deficit schizophrenia. Eur Arch Psychiatry Clin Neurosci. 2012;262(2):107–15. 10.1007/s00406-011-0224-4 21792534

[25] Pegoraro LF, Dantas CR, Banzato CE, Fuentes D. Correlation between insight dimensions and cognitive functions in patients with deficit and nondeficit schizophrenia. Schizophr Res. 2013;147(1):91–4. 10.1016/j.schres.2013.02.041 23535076

[26] Polgár P, Farkas M, Nagy O, Kelemen O, Réthelyi J, Bitter I, et al How to find the way out from four rooms? The learning of" chaining" associations may shed light on the neuropsychology of the deficit syndrome of schizophrenia. Schizophr Res. 2008;99(1–3):200–7. 17693060 10.1016/j.schres.2007.06.027

[27] Wang X, Yao S, Kirkpatrick B, Shi C, Yi J. Psychopathology and neuropsychological impairments in deficit and nondeficit schizophrenia of Chinese origin. Psychiatry Res. 2008;158(2):195–205. 10.1016/j.psychres.2006.09.007 18243336

[28] Dantas CR, Barros BR, Fernandes PT, Li LM, Banzato CE. Insight controlled for cognition in deficit and nondeficit schizophrenia. Schizophr Res. 2011;128(1–3):124–6. 10.1016/j.schres.2011.01.023 21353484

[29] Szendi I, Racsmany M, Cimmer C, Csifcsak G, Kovacs ZA, Szekeres G, et al Two subgroups of schizophrenia identified by systematic cognitive neuropsychiatric mapping. Eur Arch Psychiatry Clin Neurosci. 2010;260(3):257–66. 10.1007/s00406-009-0073-6 19842010

[30] Bombin I, Mayoral M, Castro-Fornieles J, Gonzalez-Pinto A, de la Serna E, Rapado-Castro M, et al Neuropsychological evidence for abnormal neurodevelopment associated with early-onset psychoses. Psychol Med. 2013;43(4):757–68. 10.1017/S0033291712001535 22831788

[31] Sewell RA, Perry EB Jr, Karper LP, Bell MD, Lysaker P, Goulet JL, et al Clinical significance of neurological soft signs in schizophrenia: factor analysis of the Neurological Evaluation Scale. Schizophr Res. 2010;124(1–3):1–12. 10.1016/j.schres.2010.08.036 20855185

[32] Vitzthum FB, Veckenstedt R, Moritz S. Individualized Metacognitive Therapy Program for Patients with Psychosis (MCT+): Introduction of a Novel Approach for Psychotic Symptoms. Behav Cogn Psychother. 2013:1–6. 10.1017/S1352465813000246 23631928

[33] Saleem MM, Harte MK, Marshall KM, Scally A, Brewin A, Neill JC. First episode psychosis patients show impaired cognitive function—a study of a South Asian population in the UK. J Psychopharmacol. 2013;27(4):366–73. 10.1177/0269881113477746 23427189

[34] Eack SM, Mesholam-Gately RI, Greenwald DP, Hogarty SS, Keshavan MS. Negative symptom improvement during cognitive rehabilitation: Results from a two-year trial of Cognitive Enhancement Therapy. Psychiatry Res. 2013;209(1):21–6. 10.1016/j.psychres.2013.03.020 23623449 PMC3732574

[35] Sanchez P, Pena J, Bengoetxea E, Ojeda N, Elizagarate E, Ezcurra J, et al Improvements in Negative Symptoms and Functional Outcome After a New Generation Cognitive Remediation Program: A Randomized Controlled Trial. Schizophr Bull.2013;40(3):707–15. 10.1093/schbul/sbt057 23686130 PMC3984510

[36] O'Leary DS, Flaum M, Kesler ML, Flashman LA, Arndt S, Andreasen NC. Cognitive correlates of the negative, disorganized, and psychotic symptom dimensions of schizophrenia. J Neuropsychiatry Clin Neurosci. 2000;12(1):4–15. 10678506 10.1176/jnp.12.1.4

[37] Rajji TK, Ismail Z, Mulsant BH. Age at onset and cognition in schizophrenia: meta-analysis. Br J Psychiatry. 2009;195(4):286–93. 10.1192/bjp.bp.108.060723 19794194

[38] Ekerholm M, Firus Waltersson S, Fagerberg T, Soderman E, Terenius L, Agartz I, et al Neurocognitive function in long-term treated schizophrenia: a five-year follow-up study. Psychiatry Res. 2012;200(2–3):144–52. 10.1016/j.psychres.2012.05.008 22657952

[39] Han M, Huang XF, Chen da C, Xiu MH, Hui L, Liu H, et al Gender differences in cognitive function of patients with chronic schizophrenia. Prog Neuropsychopharmacol Biol Psychiatry. 2012;39(2):358–63. 10.1016/j.pnpbp.2012.07.010 22820676

[40] Kao YC, Liu YP, Lien YJ, Lin SJ, Lu CW, Wang TS, et al The influence of sex on cognitive insight and neurocognitive functioning in schizophrenia. Prog Neuropsychopharmacol Biol Psychiatry. 2013;44:193–200. 10.1016/j.pnpbp.2013.02.006 23419242

[41] Brebion G, Villalta-Gil V, Autonell J, Cervilla J, Dolz M, Foix A, et al Cognitive correlates of verbal memory and verbal fluency in schizophrenia, and differential effects of various clinical symptoms between male and female patients. Schizophr Res. 2013;147(1):81–5. 10.1016/j.schres.2013.03.014 23578747

[42] Lin C-H, Huang C-L, Chang Y-C, Chen P-W, Lin C-Y, Tsai GE, et al Clinical symptoms, mainly negative symptoms, mediate the influence of neurocognition and social cognition on functional outcome of schizophrenia. Schizophr Res. 2013;146(1–3):231–7. 10.1016/j.schres.2013.02.009 23478155

[43] First MB, Gibbon M. User's guide for the structured clinical interview for DSM-IV axis I disorders: SCID-1 clinician version: American Psychiatric Pub; 1997.

[44] First MB, Spitzer RL, Gibbon M, Williams JBW. Structured Clinical Interview for DSM-IV Axis I Disorders: Non-patient Edition (SCID-NP). Biometrics Research Department, New York 1996.

[45] Kirkpatrick B, Buchanan RW, McKenney PD, Alphs LD, Carpenter WT Jr. The Schedule for the Deficit syndrome: an instrument for research in schizophrenia. Psychiatry Res. 1989;30(2):119–23. 2616682 10.1016/0165-1781(89)90153-4

[46] Kimhy D, Yale S, Goetz RR, McFarr LM, Malaspina D. The factorial structure of the schedule for the deficit syndrome in schizophrenia. Schizophr Bull. 2006;32(2):274–8. 16177274 10.1093/schbul/sbi064 PMC2632208

[47] Nakaya M, Ohmori K. A two-factor structure for the Schedule for the Deficit Syndrome in schizophrenia. Psychiatry Res. 2008;158(2):256–9. 10.1016/j.psychres.2007.10.008 18206248

[48] Cohen AS, Saperstein AM, Gold JM, Kirkpatrick B, Carpenter WT Jr, Buchanan RW. Neuropsychology of the deficit syndrome: new data and meta-analysis of findings to date. Schizophr Bull. 2007;33(5):1201–12. 17159230 10.1093/schbul/sbl066 PMC2632354

[49] Mueser KT, Curran PJ, McHugo GJ. Factor structure of the Brief Psychiatric Rating Scale in schizophrenia. Psychological Assess. 1997;9(3):196–204.

[50] Jaeger J, Czobor P, Berns SM. Basic neuropsychological dimensions in schizophrenia. Schizophr Res. 2003;65(2–3):105–16. 14630303 10.1016/s0920-9964(03)00052-5

[51] Kelland DZ, Lewis RF. The Digit Vigilance Test: reliability, validity, and sensitivity to diazepam. Arch Clin Neuropsychol. 1996;11(4):339–44. 14588938

[52] Schretlen DJ, Cascella NG, Meyer SM, Kingery LR, Testa SM, Munro CA, et al Neuropsychological functioning in bipolar disorder and schizophrenia. Biol Psychiatry. 2007;62(2):179–86. 17161829 10.1016/j.biopsych.2006.09.025 PMC2041824

[53] Dickinson D, Ragland JD, Gold JM, Gur RC. General and specific cognitive deficits in schizophrenia: Goliath defeats David? Biol Psychiatry. 2008;64(9):823–7. 10.1016/j.biopsych.2008.04.005 18472089 PMC4332801

[54] Cohen J. Statistical power analysis for the behavioral sciences: Routledge Academic; 1988.

[55] Rosnow RL, Rosenthal R. Computing contrasts, effect sizes, and counternulls on other people's published data: General procedures for research consumers. Psychological Med. 1996;1(1):331–40.

[56] Stevens JP. Applied Multivariate Statistics for the Social Sciences: Routledge Academic; 2012.

[57] Bora E, Yucel M, Pantelis C. Cognitive functioning in schizophrenia, schizoaffective disorder and affective psychoses: meta-analytic study. Br J Psychiatry. 2009;195(6):475–82. 10.1192/bjp.bp.108.055731 19949193

[58] Chen C, Jiang W, Zhong N, Wu J, Jiang H, Du J, et al Impaired processing speed and attention in first-episode drug naive schizophrenia with deficit syndrome. Schizophr Res. 2014;159(2–3):478–84. 10.1016/j.schres.2014.09.005 25261044

[59] Brinkman TM, Reddick WE, Luxton J, Glass JO, Sabin ND, Srivastava DK, et al Cerebral white matter integrity and executive function in adult survivors of childhood medulloblastoma. Neuro Oncol. 2012;14 Suppl 4:iv25–36. 10.1093/neuonc/nos214 PMC3480251 23095827

[60] van Schouwenburg MR, O'Shea J, Mars RB, Rushworth MF, Cools R. Controlling human striatal cognitive function via the frontal cortex. J Neurosci. 2012;32(16):5631–7. 10.1523/JNEUROSCI.6428-11.2012 22514324 PMC6703498

[61] Volpe U, Mucci A, Quarantelli M, Galderisi S, Maj M. Dorsolateral prefrontal cortex volume in patients with deficit or nondeficit schizophrenia. Prog Neuropsychopharmacol Biol Psychiatry. 2012;37(2):264–9. 10.1016/j.pnpbp.2012.02.003 22349577

[62] Buchanan RW, Breier A, Kirkpatrick B, Elkashef A, Munson RC, Gellad F, et al Structural abnormalities in deficit and nondeficit schizophrenia. Am J Psychiatry. 1993;150(1):59–65. 8417581 10.1176/ajp.150.1.59

[63] Wheeler AL, Wessa M, Szeszko PR, Foussias G, Chakravarty MM, Lerch JP, et al Further neuroimaging evidence for the deficit subtype of schizophrenia: a cortical connectomics analysis. JAMA psychiatry. 2015;72(5):446–55. 10.1001/jamapsychiatry.2014.3020 25786193

[64] Fischer BA, Keller WR, Arango C, Pearlson GD, McMahon RP, Meyer WA, et al Cortical structural abnormalities in deficit versus nondeficit schizophrenia. Schizophr Res. 2012;136(1–3):51–4. 10.1016/j.schres.2012.01.030 22336954 PMC3298625

[65] Cascella NG, Fieldstone SC, Rao VA, Pearlson GD, Sawa A, Schretlen DJ. Gray-matter abnormalities in deficit schizophrenia. Schizophr Res. 2010;120(1–3):63–70. 10.1016/j.schres.2010.03.039 20452187

[66] Sigmundsson T, Suckling J, Maier M, Williams S, Bullmore E, Greenwood K, et al Structural abnormalities in frontal, temporal, and limbic regions and interconnecting white matter tracts in schizophrenic patients with prominent negative symptoms. Am J Psychiatry. 2001;158(2):234–43. 11156806 10.1176/appi.ajp.158.2.234

[67] Hegde S, Thirthalli J, Rao SL, Raguram A, Philip M, Gangadhar BN. Cognitive deficits and its relation with psychopathology and global functioning in first episode schizophrenia. Asian J Psychiatr. 2013;6(6):537–43. 10.1016/j.ajp.2013.07.002 24309868

[68] Insel TR. Rethinking schizophrenia. Nature. 2010;468(7321):187–93. 10.1038/nature09552 21068826

[69] Palmer BW, Dawes SE, Heaton RK. What do we know about neuropsychological aspects of schizophrenia? Neuropsychol Rev. 2009;19(3):365–84. 10.1007/s11065-009-9109-y 19639412 PMC2745531

[70] Bustillo JR, Thaker G, Buchanan RW, Moran M, Kirkpatrick B, Carpenter WT Jr. Visual information-processing impairments in deficit and nondeficit schizophrenia. Am J Psychiatry. 1997;154(5):647–54. 9137120 10.1176/ajp.154.5.647

[71] Strauss GP, Allen DN, Duke LA, Ross SA, Schwartz J. Automatic affective processing impairments in patients with deficit syndrome schizophrenia. Schizophr Res. 2008;102(1–3):76–87. 10.1016/j.schres.2008.01.014 18313270

[72] Strauss GP, Jetha SS, Ross SA, Duke LA, Allen DN. Impaired facial affect labeling and discrimination in patients with deficit syndrome schizophrenia. Schizophr Res. 2010;118(1–3):146–53. 10.1016/j.schres.2010.01.016 20181462

