# Supplementary material for: Neurocognitive Impairments in Deficit and Non-Deficit Schizophrenia and Their Relationships with Symptom Dimensions and Other Clinical Variables
Source: PLoS One. 2015 Sep 18;10(9):e0138357. doi: 10.1371/journal.pone.0138357 (PMC4575183; doi:10.1371/journal.pone.0138357)
Supplement: S1 Table — Note: **p<0.001; * p<0.05. (DOCX) [file pone.0138357.s001.docx]

**Supplementary Table S1**

1. **Pearson correlation analyses among cognitive domains in DS group**

| *r* | Attention | Ideation fluency | Cognitive flexibility | Visuospatial memory |
| --- | --- | --- | --- | --- |
| Attention | - | 0.454* | 0.688** | 0.364* |
| Ideation fluency | - | - | 0.245 | 0.225 |
| Cognitive flexibility | - | - | - | 0.341* |
| Visuospatial memory | - | - | - | - |

Note: ***p*<0.001; * *p*<0.05.

1. **Pearson correlation analyses among cognitive domains in NDS group**

| *r* | Attention | Ideation fluency | Cognitive flexibility | Visuospatial memory |
| --- | --- | --- | --- | --- |
| Attention | - | 0.429* | 0.614** | 0.262* |
| Ideation fluency | - | - | 0.488** | 0.361* |
| Cognitive flexibility | - | - | - | 0.377* |
| Visuospatial memory | - | - | - | - |

Note: ***p*<0.001; * *p*<0.05.
