# Supplementary material for: Neurocognitive Impairments in Deficit and Non-Deficit Schizophrenia and Their Relationships with Symptom Dimensions and Other Clinical Variables
Source: PLoS One. 2015 Sep 18;10(9):e0138357. doi: 10.1371/journal.pone.0138357 (PMC4575183; doi:10.1371/journal.pone.0138357)
Supplement: S2 Table — Note: * p<0.05. (DOCX) [file pone.0138357.s002.docx]

**Supplementary Table S2**

1. **Pearson correlation analyses between clinical features and cognitive domains in DS group**

| *r* | Age | Education | CPZ | Course | Age at onset | BPRS Total | SAPS Total | SANS Total |
| --- | --- | --- | --- | --- | --- | --- | --- | --- |
| Attention | -0.337* | 0.107 | 0.164 | -0.301 | -0.131 | -0.070 | -0.106 | -0.498* |
| Ideation fluency | -0.101 | 0.161 | 0.072 | -0.021 | -0.203 | -0.190 | -0.063 | -0.398* |
| Cognitive flexibility | -0.163 | 0.043 | 0.151 | -0.116 | -0.132 | 0.193 | 0.001 | -0.374* |
| Visuospatial memory | -0.159 | 0.142 | 0.209 | -0.221 | 0.125 | 0.107 | 0.049 | -0.400* |

Note: * *p*<0.05.

1. **Pearson correlation analyses between clinical features and cognitive domains in NDS group**

| *r* | Age | Education | CPZ | Course | Age at onset | BPRS Total | SAPS Total | SANS Total |
| --- | --- | --- | --- | --- | --- | --- | --- | --- |
| Attention | -0.334* | 0.280* | -0.120 | -0.289* | -0.131 | -0.200 | -0.114 | -0.489** |
| Ideation fluency | -0.259 | 0.265* | -0.168 | -0.205 | -0.155 | -0.034 | 0.223 | -0.354* |
| Cognitive flexibility | -0.369* | 0.400* | -0.071 | -0.441* | 0.210 | -0.146 | -0.062 | -0.382* |
| Visuospatial memory | -0.208 | 0.262* | -0.152 | -0.140 | -0.196 | 0.095 | 0.171 | -0.265* |

Note: * *p*<0.05.
