# Supplementary material for: Neurocognitive Impairments in Deficit and Non-Deficit Schizophrenia and Their Relationships with Symptom Dimensions and Other Clinical Variables
Source: PLoS One. 2015 Sep 18;10(9):e0138357. doi: 10.1371/journal.pone.0138357 (PMC4575183; doi:10.1371/journal.pone.0138357)
Supplement: S3 Table — Note: **p<0.001; * p<0.05. (DOCX) [file pone.0138357.s003.docx]

**Supplementary Table S3**

1. **Linear regression analyses for independent contributions of the negative symptom to cognitive function in DS group**

|  | Attention | Ideation fluency | Cognitive flexibility | Visuospatial memory |
| --- | --- | --- | --- | --- |
| SANS Total | *β*=-0.498 | *β*=-0.398 | *β*=-0.374 | *β*=-0.400 |
|  | *t*=-3.538 | *t*=-2.674 | *t*=-2.483 | *t*=-2.690 |
|  | *p*=0.001* | *p*=0.011* | *p*=0.018* | *p*=0.011* |

Note: * *p*<0.05

1. **Linear regression analyses for independent contributions of education, course and the negative symptom to cognitive function in NDS group**

|  | Attention | Ideation fluency | Cognitive flexibility | Visuospatial memory |
| --- | --- | --- | --- | --- |
| Education | *β*=0.280 | *β*=0.265 | *β*=0.400 | *β*=0.262 |
|  | *t*=2.160 | *t*=2.041 | *t*=3.233 | *t*=2.010 |
|  | *p*=0.035* | *p*=0.046* | *p*=0.002* | *p*=0.049* |
| Course | *β*=-0.289 | *β*=-0.205 | *β*=-0.441 | *β*=-0.140 |
|  | *t*=-2.236 | *t*=-1.552 | *t*=-3.649 | *t*=-1.047 |
|  | *p*=0.029* | *p*=0.126 | *p*=0.001* | *p*=0.300 |
| SANS Total | *β*=-0.489 | *β*=-0.354 | *β*=-0.382 | *β*=-0.265 |
|  | *t*=-4.162 | *t*=-2.810 | *t*=-3.064 | *t*=-2.035 |
|  | *P*<0.001** | *p*=0.007* | *p*=0.003* | *p*=0.047* |

Note: ***p*<0.001; * *p*<0.05.
