# Supplementary material for: Neurocognitive Impairments in Deficit and Non-Deficit Schizophrenia and Their Relationships with Symptom Dimensions and Other Clinical Variables
Source: PLoS One. 2015 Sep 18;10(9):e0138357. doi: 10.1371/journal.pone.0138357 (PMC4575183; doi:10.1371/journal.pone.0138357)
Supplement: S4 Table — NOTE: The regression analysis was only performed in the variables correlated with the cognitive functions. (DOCX) [file pone.0138357.s004.docx]

**Supplementary Table S4**

**Comparison of the mediator role of cognitive flexibility and attention function between clinical variable and the other cognitive domains in NDS group**

A. Does cognitive flexibility fully or partially mediate the relationship between clinical variables and the other cognitive domains?

|  | Attention | Ideation fluency | Visuospatial memory |
| --- | --- | --- | --- |
| Education | fully | fully | fully |
| Course | fully | - | - |
| SANS Total | partially | fully | fully |

B. Does attention fully or partially mediate the relationship between clinical variables and the other cognitive domains?

|  | Cognitive flexibility | Ideation fluency | Visuospatial memory |
| --- | --- | --- | --- |
| Education | partially | fully | fully |
| Course | partially | - | - |
| SANS Total | fully | fully | No mediation |

NOTE: The regression analysis was only performed in the variables correlated with the cognitive functions.
